# It's Time to Say Goodbye to the ESUS Construct

**DOI:** 10.3389/fneur.2020.00653

**Published:** 2020-07-07

**Authors:** Blanca Fuentes, Raquel Gutiérrez-Zúñiga, Exuperio Díez-Tejedor

**Affiliations:** ^1^Department of Neurology and Stroke Center, Hospital Universitario La Paz, Madrid, Spain; ^2^Department of Medicine, Universidad Autónoma de Madrid, Madrid, Spain

**Keywords:** ESUS (embolic stroke of undetermined source), stroke**—**diagnosis, therapy, cryptogenic stoke, Atheroschlerosis, PFO (patent foramen ovale)

Cryptogenic stroke has been a challenge for years in clinical practice, given it can represent up to 40% of strokes, depending on the etiological classification used, which has different operative definitions for those patients in whom the complete diagnostic workup does not reveal a specific stroke etiology. In 2014, two opposing approaches to the challenge of cryptogenic stroke were proposed. On the one hand, Bang et al. proposed its assessment using advanced diagnostic techniques (such as advanced vascular imaging and cardiac monitoring, aortogenic, and paradoxical embolic source assessment, coagulopathy, and cancer screening tests) as a measure to reduce the proportion of cryptogenic strokes by increasing the diagnosis of atheroembolic disease, aortic embolic disease, branch occlusive disease, paroxysmal atrial fibrillation, paradoxical embolism, and cancer-related coagulopathy (1). On the other hand, the Cryptogenic Stroke/ESUS International Working Group proposed a new clinical construct that they named *embolic stroke of undetermined source* (ESUS) (2). The rationale behind the ESUS concept was the assumption that most cryptogenic strokes were thromboembolic and could benefit from non-vitamin K agonist oral anticoagulants (NOACs) that had already demonstrated their efficacy and safety in atrial fibrillation (AF), to reduce recurrent brain ischemia. This suggestion prompted the development of randomized clinical trials testing the safety and efficacy of rivaroxaban and dabigatran in patients with ESUS as well as the rapid dissemination of the ESUS concept to clinical practice due to the simplicity of the diagnostic workup, which required only the demonstration by neuroimaging procedures [computed tomography (CT) or magnetic resonance imaging (MRI)] of a non-lacunar infarction, the absence of extracranial or intracranial atherosclerosis causing ≥50% luminal stenosis in arteries supplying the ischemic area, no major-risk cardioembolic source of embolism and no other cause of stroke identified. The only diagnostic procedure assessments required were brain CT or MRI, 12-lead electrocardiogram (ECG), precordial echocardiography, cardiac monitoring for ≥24 h with automated rhythm detection and imaging of extracranial and intracranial arteries by either ultrasonography or MRI, CT, or catheter angiography.

However, the failure of the RESPECT-ESUS and NAVIGATE-ESUS trials (3, 4) in demonstrating any efficacy in the prevention of stroke recurrences in patients with ESUS has called into question the practical usefulness of this concept as, compared with the concept of cryptogenic stroke, the only difference is the exclusion of lacunar stroke (5–9). Therefore, ESUS remains a non-diagnosis similar to the classic cryptogenic stroke concept. Some of the most commonly used stroke classifications, such as the TOAST (10) and the ESUS criteria themselves (2), were developed for use in clinical trials; however, they have been incorporated in clinical practice without enough validation studies to truly evaluate their usefulness in clinical settings. Stroke physicians attend stroke patients daily in whom there is no clear evidence of the underlying etiology and must choose between two options. One is to make the diagnosis of ESUS (which is not actually associated with any specific therapeutic change), and the other is to make a greater effort to identify the ultimate cause of the stroke by the use of advanced diagnostic techniques (1). Choosing one or the other option is crucial for secondary stroke prevention, given the treatments can be different.

In this opinion article, we would like to highlight some of the arguments against the use of the ESUS concept in clinical practice:

- The rationale behind the ESUS concept, considering that many ESUSs would be due to covert paroxysmal AF, and therefore would benefit from the use of NOAC, has clearly failed, not only because of the neutral/negative results of the RESPECT-ESUS and NAVIGATE-ESUS trials (3, 4), but also because clinical trials with long-term ECG monitoring as well as observational studies have shown that covert AF represents only about 30% of ESUS (11–13).
  This low rate of AF detection during follow-up, the different phenotypes between patients with ESUS and patients with stroke with AF, and data from studies with implantable cardiac monitoring devices showing that the majority of embolic events do not occur proximal to episodes of AF have raised doubts regarding the causal association between covert AF and ESUS (14).
- The authors of the ESUS construct acknowledged that arteriogenic embolism due to non-stenotic plaques was possible in some patients with ESUS (2). However, grouping them into the same category as patients with other minor cardioembolic strokes, assuming that they would also benefit from anticoagulants, risks neglecting the atherothrombotic origin in patients with stroke with carotid atherosclerosis with stenosis lower than 50% or with aortic arch atheroma (AAA); these etiologies require a more tailored approach to atherosclerosis to prevent not only stroke recurrences, but also other vascular events such as myocardial infarction.
  Several studies have reported a higher prevalence of ipsilateral carotid plaques than contralateral carotid plaques in some ESUS cohorts (15, 16), and the global prevalence of carotid non-stenotic plaques in the ESUS Global Registry is as high as 79% (17). Recently published data from the NAVIGATE trial have shown that up to 40% of the patients included in that trial had carotid plaques, this being clearly more frequent on the ipsilateral side to the qualifying stroke. Interestingly, the group of patients with carotid plaques showed a strong tendency to higher frequency of stroke recurrences compared with those without carotid plaques (18).
  Similarly, AAA is a frequent finding in patients with ESUS (when screened), found in up to 28% in the ESUS Global registry (17) and in the 29% of patients who had transesophageal echocardiography (TEE) included in the NAVIGATE trial, and they showed a higher frequency of multiterritorial infarcts in neuroimaging (19). Therefore, for cases in which the stroke physician follows the ESUS criteria, which does not require TEE, many symptomatic AAA cases might be missed. Although clinical trials on the use of antithrombotic drugs in stroke prevention in AAA were inconclusive because of insufficient power calculation (19–21), these patients carry a higher risk of stroke recurrences than patients with other possible causes of cryptogenic stroke (20) and have a high burden of vascular risk factors and of coronary artery disease (19–21). Therefore, they should be instructed to strictly adhere to lifestyle modifications and risk factor interventions to reduce the overall vascular risk, instead of providing them with the uncertainty of an ESUS diagnosis.
- Patent foramen ovale (PFO) has also been included in the broad concept of ESUS (2), following the historical controversy on its pathogenic role in ischemic stroke. It has been reported to be present in up to 7.4% of patients with ESUS recruited in the NAVIGATE trial (22) and in 12.6% in the RESPECT-ESUS trial (3); however, actual rates could be underestimated, given TEE, or bubble transcranial Doppler were not required prior to inclusion in the trials. None of them showed NOAC to have any significant effect on reducing stroke recurrences. Moreover, given percutaneous PFO closure has been demonstrated to be safe and efficacious in the prevention of stroke recurrences in those patients with ischemic stroke related to large PFOs (especially when associated with atrial septal aneurysm) and no other cause of stroke (23, 24), they should be excluded from the ESUS category because the therapeutic approach is clearly different. Indeed, an update of current nomenclature and classifications systems has recently been proposed to include the specific category of PFO-associated stroke (25).
- Cancer-associated stroke is another possible underlying etiology in patients with ESUS (2) and data from the NAVIGATE trial reported a cancer diagnosis in up to 7.5% of the included patients. This value is probably an underestimate, given an exclusion criterion was a life expectancy of <6 months. A new cancer diagnosis at 11 months' follow-up was found in 1.7% (26). Patients with cancer had a higher risk of stroke recurrences than patients without cancer, without differences in ischemic stroke recurrences between the aspirin and rivaroxaban groups, although with a trend toward more major bleeds with rivaroxaban (26). Involved pathogenic mechanisms are non-bacterial thrombotic endocarditis, tumor emboli from occult cancer and a cancer-associated hypercoagulable state. Cancer-associated stroke has a very poor prognosis, with high mortality at follow-up (27, 28). Identifying and treating the underlying cancer is crucial in these patients. For this reason, following such a basic diagnostic approach as required for ESUS appears to be inappropriate for the detection of stroke-associated cancer and, in our opinion, further laboratory tests (such as D-dimer, which has been proposed as a helpful parameter for suspected covert cancer in stroke patients) (29) and cardiac examinations (such as TEE to rule out non-bacterial thrombotic endocarditis) should be performed.
- Finally, there are some other less recognized cardioembolic sources of stroke, such as atrial cardiopathy and left ventricular disease, including hypertrophy, decreased ejection fraction and valvular heart disease without AF, which merit identification in patients with stroke. The results of the ongoing ATTICUS and ARCADIA trials (30, 31) that are investigating the efficacy and safety of apixaban in patients with disease of unknown etiology and atrial cardiopathy or at least one risk factor suggestive of cardiac embolism should provide us with new insights into the role of atrial cardiopathy and the risk of stroke, whether mediated or not by covert AF. These trials are selecting cryptogenic stroke patients who present the following markers suggestive of atrial cardiopathy: left atrium enlargement >45 mm, spontaneous echo contrast in left atrial appendage (LAA), LAA flow velocity ≤ 0.2 m/sg, atrial high rate episodes, PFO and high CHADS2-VASc score (≥4) in the ATTICUS Trial; and the *P*-wave terminal force > 5,000 μV x ms in ECG lead V1, serum N-terminal pro-brain natriuretic peptide (NT-ProBNP) >250 pg/ml, and left atrial diameter index ≥3 cm/m^2^ in the ARCADIA Trial (30, 31).

Therefore, in our opinion, the exclusion of ipsilateral non-stenotic carotid plaques, aortic arch atherosclerosis, PFO, and cancer-associated strokes should be a prerequisite before diagnosing a cryptogenic stroke in clinical practice (Figure 1), and patients with factors that have been identified as being associated with a higher risk of covert AF should undergo long-term cardiac monitoring. In this sense, the evaluation of serum (Nt-ProBNP) could help in the selection of patients for long-term cardiac monitoring since levels ≥505 pg/ml have recently shown to have a 86% sensitivity and 98% negative predictive value for AF in cryptogenic stroke (32).

**Figure 1:**
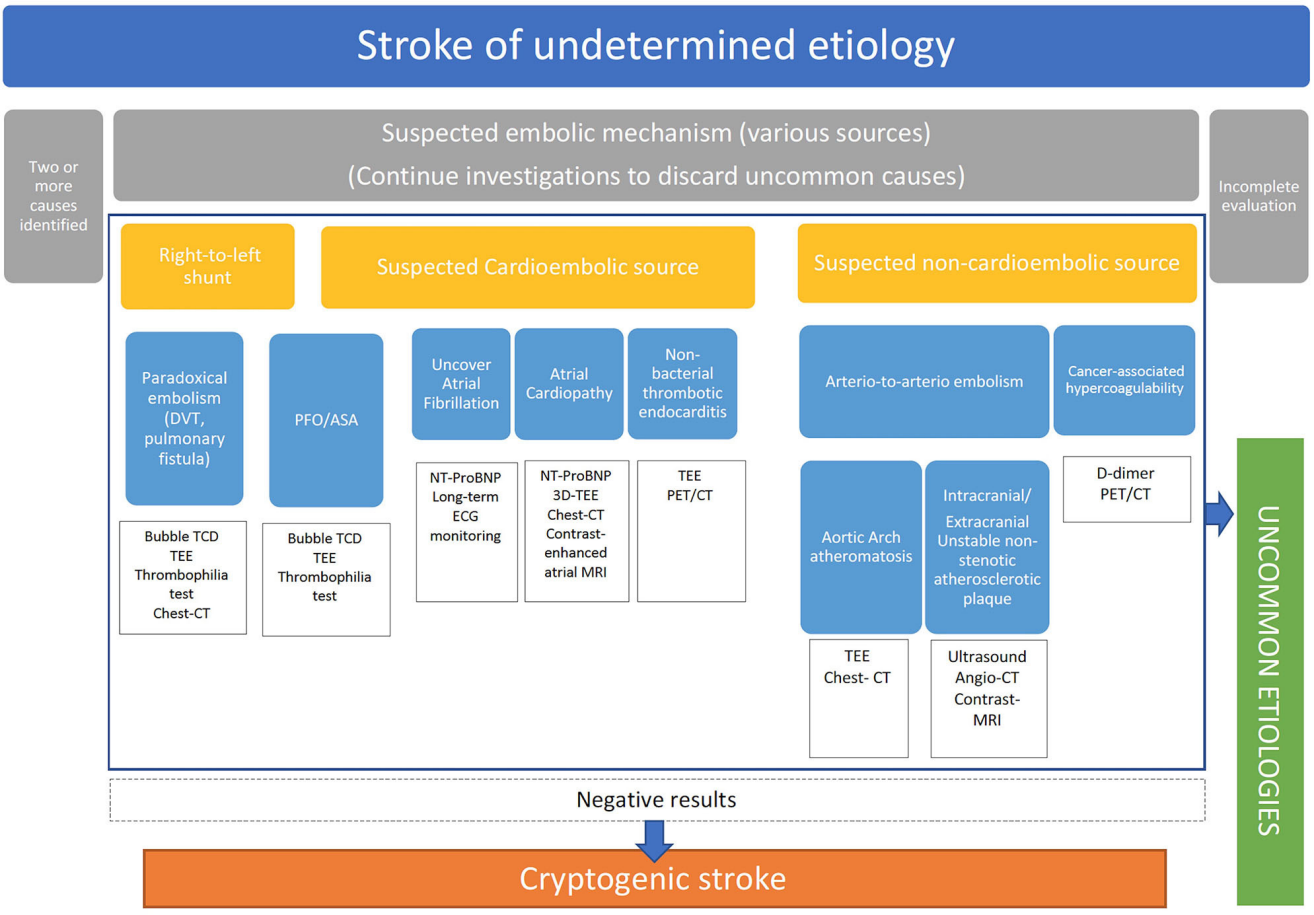
A proposal for stroke of unknown etiology, cryptogenic stroke classification and diagnostic approach. CT, Computed Tomography; DVT, Deep Venous Thrombosis; ECG, Electrocardiogram; MRI, Magnetic Resonance Imaging; PET/CT, Positron Emission Tomography/Computed Tomography; TCD, Transcranial Doppler; TEE, Transesophageal Echocardiography.

In conclusion, our advice to stroke physicians is to forget ESUS and be smart in the search for underlying causes of ischemic stroke, optimizing advanced diagnostic procedures according to the patient's and stroke's characteristics, attempting to find the correct diagnosis for stroke patients and reducing rates of cryptogenic stroke diagnosis.

## Author Contributions

All authors listed have made a substantial, direct and intellectual contribution to the work, and approved it for publication.

## Conflict of Interest

BF: Advisory Board (Bayer), speakers' honoraria (BMS-Pfizer, Daichi-Sankyo), travel grants to attend scientific meetings (Daichi-Sankyo, BMS-Pfizer), and participation in clinical trials (RESPECT-ESUS, NAVIGATE-ESUS). RG-Z: participation in clinical trials (RESPECT-ESUS, NAVIGATE-ESUS). ED-T: travel grants to attend scientific meetings (Daichi-Sankyo) and participation in clinical trials (RESPECT-ESUS, NAVIGATE-ESUS).
